# Social Network‐Based Induced Abortion Incidence Estimation in Burkina Faso: Examining the Impact of the Network Generating Question

**DOI:** 10.1111/sifp.12213

**Published:** 2022-10-09

**Authors:** Suzanne O. Bell, Georges Guiella, Selena Anjur‐Dietrich, Fiacre Bazie, Yentema Onadja, Saifuddin Ahmed, Caroline Moreau

**Affiliations:** ^1^ Department of Population, Family and Reproductive Health Johns Hopkins Bloomberg School of Public Health Baltimore MD 21205 USA; ^2^ Institut Supérieur des Sciences de la Population (ISSP) Université Joseph Ki‐Zerbo Ouagadougou Burkina Faso; ^3^ Soins Primaires et Prévention CESP Centre for Research in Epidemiology and Population Health U1018, Inserm Villejuif F‐94800 France

## Abstract

Social network‐based methods are increasingly used to estimate induced abortion incidence and investigate correlates. Approaches differ in the social tie definitions used to identify which social network members’ abortion experiences respondents will report. This study compares the effect of using the “best friend” (closest female friend) versus “confidante” (specifying mutual sharing of personal information) definition on abortion incidence estimation. We use data from a nationally representative survey of women aged 15–49 in Burkina Faso (conducted in 2020–2021) where respondents were randomized into two versions of an abortion module, using different friend definitions. We computed abortion rate estimates by friend definition and adjusted for assumption violations (transmission bias, surrogate sample selection bias). Unadjusted incidence rates varied from 11.7 [4.1–19.2] abortions per 1,000 women to 15.6 [9.7–21.4], depending on friend definition. The confidante definition yielded higher adjusted estimates (36.2 [25.1–47.2]) than the best friend definition (17.0 [8.7–25.3]) due to greater transmission bias adjustment. Both estimates exceeded the respondent self‐reported abortion incidence (4.0 [2.2–5.9]). Our results indicate that either friend definition produces higher incidence estimates than self‐report but suggest a potential advantage for the “best friend” over the “confidante” definition given lower transmission bias. Further research should assess generalizability of these findings in other contexts.

## BACKGROUND

Induced abortion is legal in Burkina Faso only in cases of rape, incest, fetal defects, or when the woman's health is in danger. The most recent national estimates based on abortion cases admitted for treatment of complications at a health facility in 2008 show a one‐year incidence rate of 25 induced abortions per 1,000 women of reproductive age, while the estimated incidence using a social network‐based approach as part of the same study was 18 per 1,000 (Sedgh et al. 2011). Subsequent declines in the total fertility rate in Burkina Faso, resulting from increasing demand for and use of modern contraception (Institut National de la Statistique et de la Démographie (INSD) et ICF International 2012; Institut National de la Statistique et de la Démographie (INSD) et al. 2018; Institut Supérieur des Sciences de la Population 2020) may have affected levels and patterns of unintended pregnancy and related abortion trends. These demographic shifts have taken place amid a changing abortion landscape, characterized by an expansion of legal grounds for abortion as well as the diffusion of medication abortion drugs outside of the health care system in many parts of Africa (Drabo 2019). These social and legal changes make it important to understand how the frequency and correlates of abortion have changed in intervening years. A recent model‐based estimate of the national abortion incidence suggests a rate of 30 (95 percent CI 18–49) abortions per 1,000; however, the wide confidence interval means we are unable to determine if the change is significant (Bearak et al. 2022). These prior incidence studies also offer little to no information concerning the safety of abortions in Burkina Faso and few if any details on the characteristics of women having an abortion, yet such data are critical to inform the public health rationale for legal reform and the provision of quality, safe abortion care for legal indications as well as postabortion care to treat unsafe abortion‐related complications, a leading cause of maternal mortality in West Africa (Say et al. 2014; Kassebaum et al. 2014).

In recent years, there have also been developments in induced abortion incidence estimation, with several studies exploring social network‐based approaches to more accurately quantify this sensitive behavior. Many of these methods have the added value of providing individual‐level data, enabling the collection of detailed information on women's characteristics for estimating abortion incidence by background characteristics, and detailed information about the abortion itself for estimating abortion safety. These approaches include the anonymous third‐party reporting (ATPR) method, best friend method, confidante method, and network‐scale up method (Rossier et al. 2006; Keogh et al. 2020; Sully, Giorgio, and Anjur‐Dietrich 2020; Bell, Shankar, et al. 2020; Yeatman and Trinitapoli 2011; Elul 2004; Sedgh and Keogh 2019; Stillman et al. 2020; Giorgio, Sully, and Chiu 2021). These methods rely on several assumptions that must be met in order to produce accurate estimates, which broadly include (1) respondents know about the abortion experiences of their close friends (i.e., no transmission bias), (2) the surrogate sample of friends resembles that of the respondents (i.e., no selection bias), and (3) social desirability pressure is reduced when reporting on the stigmatized behaviors of one's friends as opposed to oneself (Fisher 1993; Shelley et al. 1995; Feehan and Salganik 2016; McPherson, Smith‐Lovin, and Cook 2001). While these estimation approaches are similar in many regards, they vary with regard to the network generating question (i.e., how they define the relationship of interest) and the number of people from a woman's social network about which they collect data.

The relationships between respondents and third parties can be specified very broadly to generate a large surrogate sample, as in the network scale‐up method, or more narrowly defined to maximize the chance of knowing about intimate details of others’ lives (as in the best friend, confidante, and ATPR methods). The ATPR method requires that members of the surrogate sample confide in the respondent but not necessarily vice versa (Rossier 2010), while the confidante method requires reciprocal sharing of sensitive information (Sedgh and Keogh 2019). In contrast, the best friend method simply requires a close relationship without further stipulating the type of communication it comprises (Yeatman and Trinitapoli 2011). The performance of these social network‐based approaches has been mixed, with limited understanding of what aspects of the design produce the least transmission bias and most valid surrogate sample. Evidence that respondents often have few, if any, close relationships in some contexts, that information sharing between respondents degrades among their second‐ or third‐closest confidantes, and that the surrogate confidante population becomes less representative of the population of reproductive age women with higher order confidantes led some to implement the confidante method relying on only one confidante to estimate abortion incidence (Stillman et al. 2020; Bell, Shankar, et al. 2020; Keogh et al. 2020; Ahmad et al. 2020; Bell, Sheehy, et al. 2020; Bell, Omoluabi, et al. 2020; Giorgio, Sully, and Chiu 2021). In addition, findings from a previous multicountry study using the confidante definition led researchers to believe that its stringent mutual sharing of sensitive information criteria may contribute to a biased surrogate sample if respondents report on a more selective group of friends (Bell, Shankar, et al. 2020). Specifically, the criterion of mutual sharing of sensitive information may imply to the respondent that such sharing must already have occurred, potentially resulting in an overrepresentation of confidantes with sensitive or stigmatized experiences, including abortions (Giorgio, Sully, and Chiu 2021). A less strict relationship definition, as in the best friend method, may reduce this concern of friend selection based on prior abortion experiences.

In this study, we seek to formally test the performance of social network‐based methods on abortion incidence estimation by relationship definition: “confidante” relationship specifying mutual sharing of very personal information versus “best friend” relationship requiring no mutual sharing. We compare the social network‐based reporting‐related assumptions (transmission bias, representativeness of the surrogate population, and reduced social desirability bias) by relationship definition.

## METHODS

### Data

We used the Performance Monitoring for Action (PMA) survey to implement an abortion module in Burkina Faso (Performance Monitoring for Action (PMA) 2021). Fieldwork occurred from December 2020 through March 2021. The survey design employed a two‐stage urban–rural stratified cluster sampling approach with probability proportional to size sampling of geographic areas or clusters in Burkina Faso to identify a nationally representative sample of women aged 15–49 years (Performance Monitoring for Action (PMA) 2022). Interviewers created a master sampling frame of all households within each cluster, randomly selected 35, and interviewed a member of the household to create a household roster with all members’ ages and genders. The interviewer then invited all females between the ages of 15 and 49 to participate in the female questionnaire. We constructed design‐survey weights using the inverse of the household selection probability and further adjusted the weights for nonresponse at the household level within the cluster. Trained interviewers from in or near the communities being surveyed administered the questionnaire face‐to‐face via smartphone in French or a local language to women who provided verbal consent to participant. The Johns Hopkins University Bloomberg School of Public Health and the Comité d'Ethique pour la recherche en santé/ Ministère de la Santé et Ministère de l'Enseignement Supérieur, de la Recherche Scientifique et de l'Innovation in Burkina Faso provided ethical approval for the study.

### Measurement

The abortion module included both direct and indirect questions on abortion, drawing from the experience of the prior PMA abortion work in Nigeria, Cote d'Ivoire, and India (Bell, Omoluabi, et al. 2020; Bell, Shankar, et al. 2020; Bell, Sheehy, et al. 2020; Ahmad et al. 2020). Prior to any mention of abortion, respondents were randomly assigned (automatically through the Open Data Kit data collection application) to respond to questions on their closest female confidante (“confidante” method) or their closest female friend (“best friend” method) aged 15–49 and currently living in Burkina Faso. The “confidante” was identified using the following question: “Now I want to ask some questions about your closest female friends (these can be friends or relatives, like sisters, cousins, a mother, or aunts). These are women whom you share very personal information with and who also share their very personal information with you. How many female friends or relatives like this do you have in Burkina Faso who are between the ages of 15 and 49?” (French: “J'aimerais maintenant vous poser quelques questions sur vos amies femmes ou filles les plus proches (il peut s'agir d'amies ou de membres de votre famille, comme des sœurs, des cousines, mère ou tantes) à qui vous confiez vos secrets et qui vous confient les leurs. Combien d'amies de ce genre avez‐vous qui ont entre 15 et 49 ans et habitent au Burkina Faso ?”). The best friend was identified using a similar question but with no reference to mutual sharing: “Now I want to ask some questions about your female friends (these can be friends or relatives, like sisters, cousins, a mother, or aunts). How many close female friends or relatives do you have in Burkina Faso who are between the ages of 15 and 49?” (French: “J'aimerais maintenant vous poser quelques questions sur vos amies femmes ou filles les plus proches (il peut s'agir d'amies ou de membres de votre famille, comme des sœurs, des cousines, mère ou tantes). Combien d'amies proches avez‐vous qui ont entre 15 et 49 ans et habitent au Burkina Faso ?”). Regardless of friend definition, interviewers collected information on the closest friend's age, highest level of education, whether currently married or cohabiting, number of children, whether they had ever used contraception, whether they were currently using contraception, and contraceptive method type.

Abortions were identified using two questions capturing a range of experiences. The first question asked about ever doing something to end a pregnancy, while the next question asked about ever doing something to bring back a late menstrual period. For both questions, we confirmed whether it was intentional and successful, and for the period regulation question, we clarified that they took this action because they were worried about being pregnant. Further details on the abortion terminology are provided elsewhere (Bell, Omoluabi, et al. 2020; Bell and Fissell 2021; Sheehy et al. 2021). These questions, developed in PMA pilot studies in Nigeria and Cote d'Ivoire, were piloted among resident interviewers who spoke a variety of languages to assess the relevance of these terminologies in the Burkina context. We tested the draft questions and asked follow‐up questions to gauge question comprehension with 24 respondents (six conducted in French, the rest in local languages) and refined questions on abortion accordingly. Respondents who indicated they had ended a pregnancy or brought back a late period provided additional details about the most recent event, including how certain they were about being pregnant, the year it occurred, the methods used, the sources used, and whether the respondent sought care for a potential complication.

### Analyses

We conducted analyses to evaluate the performance of each assumption by friend definition. We then employed analytic methods to adjust abortion rate estimates for observed assumption violations to minimize bias introduced by these violations.

#### Assumption 1: Transmission of Abortion Knowledge

To examine whether transmission of abortion information was complete between respondents and their confidantes or best friends, we first explored differences in respondent characteristics by whether they reported having a close friend, by friend definition (confidante or best friend). We then examined patterns of respondent abortion sharing with their friend, by friend definition. Because of the complex survey design and the use of weighting, we used design‐based *F*‐tests to assess whether differences were statistically significant (Rao and Thomas 1989).

#### Assumption 2: Surrogate Sample Representativeness

To estimate population‐level measures of annual abortion rates for respondents’ friends, we must assume the surrogate sample of friends is representative of all reproductive age women in Burkina Faso. “Missing” friends corresponding to respondents who report zero friends may be systematically different from reported friends, leading to selection bias in the surrogate sample. Additionally, if friends are on aggregate different in their socioeconomic and reproductive characteristics from respondents, this may distort the representativeness of the surrogate sample. Using the respondents as a reference group, we compared friend characteristics to respondent characteristics, by friend definition. We used design‐based *F*‐tests to assess whether differences were statistically significant (Rao and Thomas 1989).

#### Assumption 3: Reduced Social Desirability Pressure When Reporting on Friend as Opposed to Self

To determine whether social desirability bias was reduced when asking respondents about their friend's or confidante's abortion experience as opposed to their own, we calculated separate one‐year incidence rates of abortion for respondents and their friends, by friend definition. To do so, we determined the number of abortions reported by respondents and separately for their best friends or confidantes since 2020. We included abortions reported in 2021 as we wanted to minimize the risk of displacement across years (Giorgio, Sully, and Chiu 2021). We divided the number of abortions by the number of women in that sample and the average number of years covered from January 1, 2020, through the date of the interview in early 2021 (1.06 years). Lastly, we multiplied the values by 1,000 to produce one‐year abortion rates per 1,000 women aged 15–49. We used design‐based *F*‐tests to assess whether differences between respondent and unadjusted friend abortion rates were statistically significant (Rao and Thomas 1989).

#### Adjusting for Assumptions Violations

To account for transmission bias (Assumption 1) and selection bias in the surrogate sample (Assumption 2), we made the following adjustments separately for friend and confidante samples. First, we multiplied the friend/confidante abortion rate by the inverse of the proportion of respondents who shared their abortion experience with their friend/confidante (Assumption 1) (Keogh et al. 2020; Stillman et al. 2020; Giorgio, Sully, and Chiu 2021). This adjustment assumes friends/confidantes share their abortion experience with respondents in the same way as respondents do with their friends/confidantes. Sharing of one's abortion differed by whether it was reported as ending a pregnancy or bringing back a late period, thus we used the relative contribution of each to calculate the transmission bias adjustment.

The other adjustment accounts for the fact that women in the underlying population with no friends/confidantes are excluded using this method. We thus incorporated the respondents who reported having no close friends/confidantes into our surrogate friend samples to ensure a more complete, representative surrogate sample. Given known concerns about underreporting one's abortion, we did not rely on these respondents' self‐reported abortion data. Instead, we predicted the probability of abortion for these “missing” friends (i.e., respondents with no social network) by first running a Poisson model regressing the indicator variable for whether the respondent's friend or confidante reported an abortion in the prior year on the socioeconomic and reproductive characteristics of the women in the surrogate sample (age, education, marital status, residence, wealth tertile, and whether they have any children). We then used the model to predict the likelihood that the respondents without friends/confidantes had an abortion in the prior year. We combined this predicted likelihood of abortion data for the respondents with no friends/confidantes with the existing friend/confidante abortion data to estimate the one‐year incidence of abortion in each surrogate sample. The lack of significant differences in abortion rates between respondents who reported having or not having close friends/confidantes supports the use of such a hybrid surrogate sample. Additionally, we included the friend/confidante abortions that respondents reported with some uncertainty (response option “Yes, I think so”) to further adjust for incomplete abortion visibility.

To address remaining evidence of selection bias in each surrogate sample, we constructed poststratification weights for the adjusted best friend and the confidante sample so the distribution of characteristics would more closely resemble the respondent characteristics. To do so, we reshaped the data to long form and regressed whether the woman was a friend (vs. a respondent) on socioeconomic and reproductive characteristics (age, education, marital status, residence, and parity). We then used these models to predict the likelihood of being a best friend or a confidante versus a respondent and multiplied the inverse of this probability by the survey design weight for the corresponding respondent. We ran this model separately by friend definition, producing separate poststratification weights for the adjussurrogate confidante and best friend samples.

We compared the respondent one‐year abortion incidence to the best friend or confidante incidence adjusted for transmission bias (Assumption 1) and adjusted for transmission bias and surrogate sample distortion (Assumptions 1 and 2). We compared the fully adjusted friend estimates to respondent rates by assessing whether confidence intervals overlapped given no suitable statistical test was found due to the post hoc nature of the transmission bias adjustment described above.

We conducted all analyses in Stata version 15.1 and applied survey design weights to account for the complex sampling design and calculated robust standard errors to account for clustering and weighting.

## RESULTS

The final sample included 6,388 reproductive aged women who completed the female survey. Table 1 displays respondent characteristics by the network generating question they were randomly assigned (best friend or confidante), and within these groups, by whether they reported having at least one friend. Results indicate randomization was effective as respondent characteristics were similar across groups. A similar proportion of women reported having a best friend or a confidante (78.2 percent vs. 79.8 percent). Regardless of friend definition, respondents who reported no friends were consistently different from those who did: they were more likely to be currently married/cohabiting, while those who reported 0 confidantes specifically were significantly more likely to have never attended school, and those who reported having 0 best friends were significantly older and less likely to be Catholic. Respondents with and without best friends/confidantes were similar with regard to wealth, residence, parity, current contraceptive use, and current long‐acting reversible contraceptive (LARC) use.

**TABLE 1:** Characteristics of female respondents aged 15–49 in Burkina Faso, overall and by number of female confidantes or best friends (i.e., “friends”) reported^a^

								Network generating question: confidante						Network generating question: best friend					
		All respondents		0 Friends		≥ 1 Friends		All respondents		0 Friends		≥ 1 Friends		All respondents		0 Friends		≥ 1 Friends	
		%	*N*	%	*N*	%	*N*	%	*N*	%	*N*	%	*N*	%	*N*	%	*N*	%	*N*
Age																			
	15–19	20.6	1350	**17.5**	249	**21.3**	1088	20.9	686	17.4	132	21.7	548	20.4	663	**17.6**	117	**21.0**	540
	20–29	33.5	2232	**31.0**	403	**34.1**	1815	34.3	1115	34.0	218	34.3	892	32.5	1113	**27.8**	185	**34.0**	923
	30–39	28.0	1750	**29.5**	380	**27.7**	1361	27.3	858	27.2	184	27.4	672	28.7	890	**31.8**	196	**28.1**	689
	40–49	17.9	1055	**22.1**	269	**16.8**	777	17.5	523	21.4	145	16.6	376	18.4	532	**22.8**	124	**17.0**	401
Education																			
	Never	57.5	2682	**62.8**	615	**56.0**	2042	56.9	1321	**63.5**	328	**55.0**	983	58.1	1359	62.0	287	57.0	1059
	Primary	18.2	1299	**19.1**	299	**18.1**	993	18.4	652	**19.0**	166	**18.4**	485	18.0	645	19.1	133	17.8	508
	Secondary/higher	24.3	2405	**18.1**	385	**25.9**	2007	24.7	1209	**17.5**	185	**26.6**	1020	24.0	1193	18.8	200	25.2	987
Marital status																			
	Currently married/cohabiting	75.4	4265	**78.1**	924	**74.7**	3309	75.3	2120	**79.2**	485	**74.2**	1624	75.4	2139	**77.1**	439	**75.2**	1685
	Divorced or separated/widowed	3.3	304	**4.9**	77	**2.9**	226	3.2	154	**4.3**	44	**3.0**	110	3.3	150	**5.5**	33	**2.8**	116
	Never married	21.3	1819	**17.0**	300	**22.4**	1507	21.5	908	**16.6**	150	**22.8**	754	21.2	910	**17.5**	150	**22.0**	753
Religion of household																			
	Muslim	58.9	2638	57.8	523	58.9	2093	56.9	1297	57.0	279	56.8	1012	60.7	1337	**58.6**	244	**60.8**	1081
	Catholic	25.7	1135	21.7	185	26.7	945	26.4	560	24.7	96	26.9	463	25.0	573	**18.6**	89	**26.6**	482
	Protestant	7.2	287	8.7	57	7.0	229	7.7	156	9.1	28	7.4	127	6.8	131	**8.3**	29	**6.6**	102
	Traditional or other	8.2	223	11.8	61	7.5	162	9.0	122	9.2	28	9.0	94	7.4	101	**14.5**	33	**6.0**	68
Wealth tertile																			
	Poorest	34.2	1183	30.4	220	34.9	947	34.0	590	31.1	109	34.6	477	34.4	590	*29.6*	111	35.1	470
	Middle wealth	31.8	1318	30.8	256	31.9	1049	31.7	652	33.5	146	31.2	501	31.9	665	*28.1*	110	32.7	548
	Wealthiest	34.0	3887	38.8	825	33.2	3046	34.3	1940	35.4	424	34.2	1510	33.8	1944	*42.3*	401	32.2	1536
Residence																			
	Rural	77.8	2615	76.4	523	77.9	2063	77.6	1298	75.2	265	78.1	1024	77.9	1313	77.8	258	77.7	1039
	Urban	22.2	3773	23.6	778	22.1	2979	22.4	1884	24.8	414	21.9	1464	22.1	1886	22.2	364	22.3	1515
Parity																			
	0	23.2	1875	19.7	319	24.1	1546	23.5	948	19.4	166	24.7	779	23.0	926	20.0	153	23.6	767
	1‐2	24.7	1729	24.9	349	24.7	1369	25.0	859	27.4	179	24.3	677	24.4	868	22.2	170	25.1	692
	3‐4	21.8	1409	23.9	323	21.3	1075	20.9	694	22.2	179	20.4	508	22.7	713	25.8	144	22.2	567
	5+	30.3	1372	31.5	309	29.9	1052	30.6	680	30.9	155	30.7	524	29.9	690	32.0	154	29.1	528
Currently using contraception																			
	No	67.7	3998	71.0	882	66.7	3081	67.6	1988	70.9	468	66.7	1508	67.7	2007	71.1	414	66.7	1573
	Yes	32.3	2390	29.0	419	33.3	1961	32.4	1194	29.1	211	33.3	980	32.3	1192	28.9	208	33.3	981
Currently using LARC																			
	No	86.1	5490	87.0	1130	85.8	4319	86.1	2734	87.3	591	85.8	2129	86.1	2751	86.8	539	85.8	2190
	Yes	13.9	898	13.0	171	14.2	723	13.9	448	12.8	88	14.2	359	13.9	448	13.2	83	14.2	364
Unadjusted abortion incidence		4.3	42	4.9	9	4.2	33	3.2	16	2.2	1	3.5	15.0	5.3	26.0	7.7	8.0	4.8	18.0
Total		100.0	6388	100.0	1301	100.0	5042	100.0	3182	100.0	679	100.0	2488	100.0	3199	100.0	622	100.0	2554

^a^Estimates weighted, Ns un; bold indicates *p*‐value for design‐based F test comparing respondents with 0 to 1+ friends less than 0.05.

In Table 2, we present the unadjusted and adjusted friend characteristics by friend definition and compare them to the respondent. Compared to respondents, the combined group of best friends and confidantes generally had slightly higher levels of education, were more likely to live in urban areas, and were more likely to be nulliparous, but were equally likely to currently be using any contraception, or LARC specifically. Respondents who received the confidante and best friend questions were equally likely to report a friend (approximately 68 percent) versus a relative (approximately 32 percent) (estimates not shown). After adjustments, differences in education and marital status disappeared but other differences remained, albeit all within three percentage points except for urban/rural residence.

**TABLE 2:** Characteristics of female respondents aged 15–49 and their closest female friends aged 15 to 49 in Burkina Faso by network generating question^a^

				Friends combined				Network generating question: confidante				Network generating question: best friend			
		Respondent		Unadjusted		Adjusted		Unadjusted		Adjusted		Unadjusted		Adjusted	
		%	*N*	%	*N*	%	N	%	N	%	N	%	*N*	%	*N*
Age															
	15–19	20.6	1350	20.2	983	20.4	1,250	20.5	492	20.7	633	19.8	491	20.2	616
	20–29	33.5	2232	34.5	1,692	34.8	2,127	34.2	833	35.3	1066	34.8	859	34.1	1057
	30–39	28.0	1750	27.7	1,428	27.2	1,837	26.4	693	25.8	891	29.0	735	28.5	944
	40–49	17.9	1055	17.6	884	17.7	1,174	18.9	439	18.2	592	16.4	445	17.2	582
Education															
	Never	57.5	2682	**57.1**	2,104	**57.7**	2,749	**56.2**	1034	56.9	1373	**57.9**	1070	58.4	1374
	Primary	18.2	1299	**14.3**	809	**16.5**	1124	**14.7**	397	16.7	569	**14.0**	412	16.2	553
	Secondary/higher	24.3	2405	**28.6**	2,112	**25.9**	2,513	**29.1**	1051	26.4	1240	**28.1**	1061	25.3	1270
Currently married		75.4	4265	**73.7**	3369	**74.1**	4325	**71.9**	1637	**73.2**	2133	75.5	1732	75.0	2186
Residence															
	Rural	77.8	2615	**73.4**	2045	**74.3**	2597	**73.6**	1017	**74.2**	1291	**73.2**	1028	**74.4**	1302
	Urban	22.2	3773	**26.6**	2997	**25.7**	3791	**26.4**	1471	**25.8**	1891	**26.8**	1526	**25.6**	1897
Parity															
	0	23.2	1875	**26.9**	1610	**25.1**	1939	**27.3**	811	**25.3**	980	**26.6**	799	**25.0**	958
	1+	76.8	4510	**73.1**	3429	**74.9**	4446	**72.7**	1675	**74.7**	2201	**73.4**	1754	**75.0**	2239
Currently using contraception		32.3	2390	32.5	1789	31.8	2218	29.4	856	29.4	1070	35.6	933	34.2	1144
Currently using LARC		13.9	898	15.5	829	15.0	1004	13.4	370	13.3	459	17.6	459	16.7	543
Total		100.0	6388	100.0	5042	100.0	6388	100.0	2488	100.0	3182	100.0	2554	100.0	3199

^a^ Estimates weighted, Ns unweighted; bold indicates p‐value for design‐based *F* test (reference corresponding respondents) less than 0.05.

Next, we examined respondents’ sharing patterns of their own abortions with their friends by abortion question (ending a pregnancy or bringing back a late period) and friend definition (Table 3). Sharing was more common with best friends than confidantes (72.0 percent versus 49.6 percent), although the difference was not statistically significant given the small sample of respondents reporting an abortion and a friend/confidante (*n* = 79). Women who reported their abortion as “ending a pregnancy” were more likely to share their experience with their friend (77.6 percent) than those who reported “bringing back a late period” (54.6 percent). Using the percent sharing for ending a pregnancy and period regulation and the relative distribution of these events in the prior year by friend definition (38.2 percent and 29.6 percent of abortions reported asending a pregnancy among confidantes and best friends, respectively), we calculated a transmission bias adjustment factor of 2.33 for the confidante sample and 1.37 for the best friend sample. For the combined sample of friends and confidantes, the adjustment factor was 1.64.

**TABLE 3:** Among respondents who reported an abortion and having a confidante or best friend, percentage who shared it with their friend, overall and by abortion type, network generating question, and background characteristics ^a^

								Network generating question			
		Combined		Ending pregnancy		Period regulation		Confidante		Best friend	
		%	*N*	%	*N*	%	*N*	%	*N*	%	*N*
Age											
	15–19	48.0	8	50.1	6	39.6	2	44.2	4	50.5	4
	20–29	60.4	35	89.8	14	49.8	22	62.7	18	59.0	17
	30–39	52.5	25	60.6	10	50.8	15	30.4	13	79.8	12
	40–40	89.2	11	90.9	4	87.0	7	85.2	6	91.7	5
Education											
	Never	64.8	24	100.0	8	52.7	16	40.4	13	84.1	11
	Primary	62.6	16	42.8	7	73.3	9	69.0	9	52.8	7
	Secondary/higher	56.0	39	64.5	19	51.0	21	56.6	19	55.6	20
Marital status											
	Currently married/cohabiting	65.8	47	88.0	17	58.2	30	47.7	24	80.2	23
	Divorced or separated/widowed	36 .8	6	0.0	2	49.8	4	44.8	5	0.0	1
	Never married	51 .6	26	67.7	15	32.6	12	59.1	12	45.9	14
Wealth tertile											
	Poorest	82.8	12	100.0	4	77.4	8	84.1	7	82.2	5
	Middle wealth	65.9	9	100.0	2	50.8	7	47.3	6	100.0	3
	Wealthiest	46.0	58	62.5	28	36.7	31	36.5	28	55.7	30
Residence											
	Rural	66.6	27	87.1	7	60.2	23	47.9	12	79.3	15
	Urban	52.5	52	68.7	27	38.7	23	52.1	29	53.1	23
Total		61.8	79	77.6	34	54.6	46	49.6	41	72.0	38

^a^ Estimates weighted, Ns unweig; bold indicates *p*‐value for design‐based *F* test less than 0.05.

Lastly, we present the one‐year induced abortion incidence rates for respondents and for friends by friend definition, including unadjusted and adjusted friend estimates (Table 4). The respondent one‐year incidence of abortion was 4.0 (95 percent CI 2.2–5.9) abortions per 1,000 women of reproductive age while the unadjusted rate was 15.6 (95 percent CI 9.7–21.4) for confidantes, and 11.7 (95 percent CI 4.1–19.2) for best friends. After applying the transmission bias adjustment, the confidante abortion incidence rose to 36.3 (95 percent CI 22.5–50.0) abortions per 1,000 and the best friend abortion incidence rose to 16.0 (95 percent CI 5.7–26.3). Incorporating the adjustment for nonrepresentativeness of the surrogate sample (Assumption 2), the final confidante abortion incidence rate was 36.2 (95 percent CI 25.1–47.2) while the final best friend rate was 17.0 (95 percent CI 8.7–25.3). As the transmission bias adjustment factor was not statistically significantly different by friend definition and the sample size was small, we also applied an average transmission bias adjustment factor for both confidante and best friend samples, resulting in a confidante abortion incidence rate of 25.5 (95 percent CI 17.7–33.2) and best friend incidence rate of 20.4 (95 percent CI 10.5–30.3) (estimates not shown). The unadjusted best friend and confidante abortion incidence estimates were statistically significantly higher than the respondent estimate. No valid statistical significance test was identified for comparing unadjusted respondent rates to adjusted best friend and confidante rates but given the nonoverlapping confidence intervals we interpret these estimates as significantly different.

**TABLE 4:** One‐year induceabortion incidence (per 1,000) for female respondents aged 15–49 and their closest female friends age 15 to 49 in Burkina Faso in 2020 by adjustment for biases and network generating question

							Network generating question					
	Respondent			Friend combined			Confidante			Best friend		
	Estimate	95% CI		Estimate	95% CI		Estimate	95% CI		Estimate	95% CI	
Unadjusted	4.0	2.2	5.9	**13.6**	8.2	19.0	**15.6**	9.7	21.4	**11.7**	4.1	19.2
+ adjustment for transmission bias	–	–	–	22.3	13.5	31.2	36.3	22.5	50.0	16.0	5.7	26.3
+ Poisson adjustment for missing friends	–	–	–	22.9	15.8	30.0	36.2	25.1	47.2	17.0	8.7	25.3

*Estimates weighted; bolding indicates *p*‐value for design‐based *F* test less than 0.05. No appropriate statistical significance test was identified for comparing unadjusted respondent rates to adjusted friend rates; however, since the unadjusted friend rates (combined and by friend definition) are each significantly different than respondents, and all adjustments increased the estimates and 95% CI bounds, we can infer statistically significantly higher adjusted friend rates (compared to the unadjusted respondent rate).

## DISCUSSION

Results from this study demonstrate the significance of the network‐generating question in the implementation of social network‐based approaches to measuring sensitive behaviors, as we produced a range of induced abortion incidence estimates depending on which criteria respondents considered in selecting their close friends: best friends or confidantes. Among respondents’ confidantes, we estimated a fully adjusted induced abortion incidence of 36.2 abortions per 1,000 women (95 percent CI 25.1–47.2), while among respondents’ best friends, the rate was 17.0 induced abortions per 1,000 women (95 percent CI 8.7–25.3). We were unable to directly test statistical significance of the difference between unadjusted respondent rates and the adjusted friend rates, combined and by friend definition. However, both friend rates were significantly higher than the respondent rate, based on nonoverlapping 95 percent CIs. We also found a substantial difference between best friend and confidante adjusted estimates with CIs hardly overlapping.

As is the case with nearly all induced abortion studies in legally restrictive settings, we have no way to validate our incidence estimate given we lack an external, objective measure. The most recent one‐year induced abortion incidence estimate from country‐level data, which used a different indirect estimation technique that relied on adjusting postabortion care complication data, produced an estimate of 25 abortions per 1,000 women of reproductive age in 2008 (Sedgh et al. 2011), while a recent model‐based estimate suggests a rate of 30 (95 percent CI 18–49). These estimates are between the confidante and best friend estimates and at the edge of these estimates’ confidence intervals, providing additional data points for triangulation across estimation techniques.

In this implementation of two friend definitions in Burkina Faso, neither definition demonstrates a clear advantage based on results of assumption evaluations. Respondents’ best friends and confidantes produced comparably representative surrogate samples, and similar proportions of respondents reported having at least one friend meeting either definition. Though not statistically significantly different in this study, the higher level of abortion information sharing with a best friend relative to a confidantes suggests a slight advantage of the best friend definition as it reduces the level of transmission bias adjustment, which is the most significant adjustment factor to surrogate sample abortion estimates. Further research including a larger sample of respondents reporting an abortion is needed to confirm this potential advantage observed in Burkina Faso and investigate the implications of different friend definitions in other contexts.

This study, and the social network‐based methodologies at large, has several limitations. As previously mentioned, we were unable to validate these estimates and definitively determine which friend definition produces a more accurate estimate of induced abortion. Given concerns about underreporting of abortion from self‐report data (Jones and Kost 2007), we believe indirect estimates based on friend estimates are closer to the true abortion incidence than the self‐report estimate. However, sources of bias in the social network‐based techniques remain (Giorgio, Sully, and Chiu 2021). Researchers have raised concerns about the representativeness of the surrogate sample given differences in respondent and friend characteristics even after adjustment in other settings (Giorgio, Sully, and Chiu 2021). In our research, we found these differences were small in magnitude in Burkina Faso and other settings (Bell, Shankar, et al. 2020), even when statistically significant. Another point of debate is the use of Poisson regression to account for “missing‘ friends,” which departs from perfect homophily (Giorgio, Sully, and Chiu 2021). However, we do not believe one‐to‐one homophily between respondents and friends is a requirement of the methodology and apply the analytical approach used by others when estimating mortality via sibling reports to estimate the abortion rate accounting for “missing” friends (Gakidou, and King 2006). This adjustment had little impact on our estimates given similar abortion rates among respondents with or without friends.

Other study limitations affect our adjustment for transmission bias, which we view as the more challenging aspect of this methodology. We calculated the adjustment factors based on abortion visibility within a very small sample of respondents who self‐reported having had an abortion (overall *n* = 79), further divided by friend definition, limiting the precision of our transmission bias estimate and our ability to detect differences by friend definition. If we assume the differences in transmission bias were random and apply an average transmission bias adjustment across confidante and friend data, the adjusted abortion incidence estimates are much closer – 25.4 and 20.3 for confidantes and best friends, respectively. Thus the accuracy of the abortion transmission bias has substantial impact on our final estimates. Further, if women who report their own abortion on a face‐to‐face survey differ systematically in terms of their willingness to share their abortion experience with their friend from those who do not report their abortion, the adjustment factor would be biased. However, we do not expect these potential biases to differ by friend definition.

This study also has several strengths. We randomized individuals into two network‐generating question groups, increasing the internal validity of our findings, while using a representative population‐based survey, which increases the external validity of the study. In addition, our analytic approach enabled us to verify how adjustments addressing violations of assumptions (transmission bias, selection bias) varied by friend definition. In particular, we found the use of best friend definition reduced transmission bias and the extent of the adjustment.

## CONCLUSION

Our findings offer an assessment of a key dimension of social network‐based data collection efforts for estimation of induced abortion incidence and other sensitive behaviors. The results indicate that either friend definition produces higher incidence rates than direct estimates and suggest a potential advantage for using a “best friend” over a “confidante” definition to reduce the level of adjustment due to violation of the visibility method assumption. Further research is needed to assess the generalizability of these findings in other contexts and to provide a more robust assessment of transmission bias using a larger sample size of people who report having had an abortion. As use of social network‐based approaches to measuring abortion, and sensitive behaviors more broadly, has increased in recent years, additional insights into these methods’ performance will improve the validity and value of future studies that employ these techniques. Given that most of these approaches produce individual‐level data, refining these methods—especially our understanding of transmission bias—presents a significant opportunity to know more about those who experience these stigmatized outcomes. Such findings can help inform context‐specific public health policies and programs.

## CONFLICT OF INTEREST

All authors declare that they have no conflicts of interest.

## ETHICS APPROVAL

The Johns Hopkins Bloomberg School of Public Health and the Comité d'Ethique pour la recherche en santé/ Ministère de la Santé et Ministère de l'Enseignement Supérieur, de la Recherche Scientifique et de l'Innovation in Burkina Faso provided ethical approval for the study.

## FUNDING

Funding for this study was provided by an Anonymous Donor under grant number 127941 and the Bill & Melinda Gates Foundation under grant number OPP10709004. Funders were not involved in any aspect of the study design, data collection and analysis, nor interpretation and writing of the manuscript.

## Data Availability

Data for this study are publicly available at pmadata.org. We relied on the Burkina Faso Phase 2 female dataset. Anyone can access these data after completing a brief request form at https://www.pmadata.org/data/available‐datasets.
